# Tyrosine motifs are required for prestin basolateral membrane targeting

**DOI:** 10.1242/bio.201410629

**Published:** 2015-01-14

**Authors:** Yifan Zhang, Iman Moeini-Naghani, JunPing Bai, Joseph Santos-Sacchi, Dhasakumar S. Navaratnam

**Affiliations:** 1 Department of Neurology, Yale School of Medicine, New Haven, CT 06510, USA; 2 Department of Neurobiology, Yale School of Medicine, New Haven, CT 06510, USA; 3 Department of Surgery, Yale School of Medicine, New Haven, CT 06510, USA; 4 Department of Cellular and Molecular Physiology, Yale School of Medicine, New Haven, CT 06510, USA

**Keywords:** Golgi, Hair cell, Tyrosine, Cell polarity, Protein sorting

## Abstract

Prestin is targeted to the lateral wall of outer hair cells (OHCs) where its electromotility is critical for cochlear amplification. Using MDCK cells as a model system for polarized epithelial sorting, we demonstrate that prestin uses tyrosine residues, in a YXXΦ motif, to target the basolateral surface. Both Y520 and Y667 are important for basolateral targeting of prestin. Mutation of these residues to glutamine or alanine resulted in retention within the Golgi and delayed egress from the Golgi in Y667Q. Basolateral targeting is restored upon mutation to phenylalanine suggesting the importance of a phenol ring in the tyrosine side chain. We also demonstrate that prestin targeting to the basolateral surface is dependent on AP1B (μ1B), and that prestin uses transferrin containing early endosomes in its passage from the Golgi to the basolateral plasma membrane. The presence of AP1B (μ1B) in OHCs, and parallels between prestin targeting to the basolateral surface of OHCs and polarized epithelial cells suggest that outer hair cells resemble polarized epithelia rather than neurons in this important phenotypic measure.

## INTRODUCTION

The cochlear amplifier is responsible for the exquisite sensitivity of mammalian hearing (Davis, 1983). There is considerable experimental data implicating electromotility of outer hair cells as integral to this process (Ashmore, 1987; Brownell et al., 1985; Dallos and Evans, 1995; Geisler, 1993; Geisler and Sang, 1995; Russell and Nilsen, 1997; Santos-Sacchi, 2003). Electromotility in outer hair cells is brought about by prestin, a transmembrane protein of the SLC26 family (Zheng et al., 2000), and molecular evidence has now confirmed its importance to cochlear amplification (Gao et al., 2007; Liberman et al., 2002; Mellado Lagarde et al., 2008). The localization of prestin along the lateral wall of these elongated cylindrical cells is critical to electromotility (Dallos et al., 1991; Hallworth et al., 1993; Huang and Santos-Sacchi, 1993; Kalinec et al., 1992; Yu et al., 2006; Zheng et al., 2000). The presence of prestin along the lateral wall of the cell brings about the voltage mediated elongation and shortening of outer hair cells along its longitudinal axis. How prestin is targeted to the lateral wall of the cell has been indeterminate.

Hair cells are specialized epithelial cells that show features of both epithelial cells as well as neurons. Individual cells form apically located tight junctions with other cells and have apically located stereocilia that are analogous to apically located microvilli (Leonova and Raphael, 1997; Mahendrasingam et al., 1997). Hair cells resemble neurons in containing unstable membrane potentials that result from a plethora of voltage and mechanically sensitive ion channels. These channels are sharply segregated in the cell with mechanically sensitive channels located in stereocilia (Fettiplace, 2009). In contrast many of its voltage and ligand gated ion channels are located at the basolateral surface of the cell (Housley et al., 2006). Inner hair cells, in addition, have synaptic apparatus that is located at it basal pole (Glowatzki et al., 2008).

A large body of work in polarized epithelial cells has shown the segregation of proteins to the basolateral and apical ends of the cell that results in a segregation of function (Farr et al., 2009; Rodriguez-Boulan et al., 2005). This segregation of proteins occurs by sorting of proteins after exit from the Golgi. Since neurons demonstrate a similar segregation of function, it has been proposed that dendritic and axonal compartments are analogous to the basolateral and apical surface respectively of polarized epithelial cells (Bradke and Dotti, 1998; Dotti et al., 1991; Dotti and Simons, 1990; Pietrini et al., 1994). Hair cells, however, pose a dilemma since they have features of both epithelial cells and neurons. Critically, the dendritic and axonal ends of a hair cell are at opposite ends to the expected basolateral and apical ends of the cell. Thus mechanosensitive channels that serve as its receptors are present in stereocilia and not at the basolateral surface as would be expected by its dendritic extrapolation. Similarly, the synaptic apparatus of inner hair cells is located at the basal pole and not at the stereociliary apical end as would be expected by its axonal extrapolation.

A collation of previous experimental data would suggest that protein sorting in hair cells resembles that of polarized epithelial cells rather than neurons. Thus, prior work has shown the basolateral localization of a number of proteins that are classically sorted to the basolateral surface of polarized epithelial cells. These proteins include E-cadherin, β-catenin and Na/K ATPase in mammalian, chicken and zebrafish hair cells (Bian et al., 2011; Clemens Grisham et al., 2013; Leonova and Raphael, 1997; Mahendrasingam et al., 1997). Moreover, AP1B (μ1B), a protein subunit in the AP1B clathrin protein complex integral to the basolateral sorting apparatus, is present in hair cells and its loss is important for the sorting of Na/K ATPase to the basolateral surface of hair cells (Clemens Grisham et al., 2013). This protein subunit, which was identified as critical to hair cell function in a forward screening of hair cell dysfunction mutants in zebrafish, is normally present only in epithelial cells and not in other cell types including neurons (Ohno et al., 1999).

In this paper we demonstrate that prestin, the protein responsible for outer hair cell electromotility, is localized along the basolateral surface of polarized epithelial cells using a tyrosine signal motif that is dependent on the AP1B (μ1B) subunit. These data further support an emerging concept that in hair cells protein sorting resembles that of epithelial cells rather than neurons. Previous work in other polarized CL4 epithelial cells have shown a similar sorting of two proteins, Espin and myosin Xa, associated with the stereocilliary apparatus to the apical surface of the cell and prestin to the basolateral surface of the cell. The implications to this concept are significant.

## MATERIALS AND METHODS

### cDNA constructs and generation of mutants

Single or multiple amino acid substitutions were generated using QuickChange II or QuickChange II Multi site-directed mutagenesis kits (Stratagene, La Jolla, CA) with a gerbil prestin-YFP in pEYFPN1 vector (Clontech, Mountain View, CA) as a template. All mutations were confirmed by DNA sequencing, including the entire coding region.

### Antibody labeling

Cells were fixed in 4% formaldehyde, washed in PBS 0.05% Tween 20, 0.05% Triton-X 100 (incubation buffer) three times, and incubated with primary antibody in incubation buffer overnight at 4°C. The primary antibodies were goat anti-prestin antibody (1:500, N-20 prestin, Santa Cruz, CA) mouse anti-β-catenin (1:50, Becton Dickinson), mouse anti-Na/K ATPase (1:20, Affinity Bioreagents), and goat anti-AP1B (µ1B) (1:100, Santa-Cruz). After three washes in incubation buffer the cells/tissue was incubated with secondary antibody in incubation buffer for 1 hour at room temperature. Secondary antibodies used included goat anti-mouse Alexa 647 (1:200, Becton Dickinson) and Donkey anti-goat (Beckton-Dickinson, 1:200). Actin was detected using Phalloidin Alexa 546 (1:200, Beckton-Dickinson) added to the secondary antibody. Cells were washed in incubation buffer mounted in Vectashield and viewed using a Zeiss 510 laser scanning microscope.

### Cochlea processing

Tissue from 3+ week old mice (C57BL/6) were obtained after animals were euthanized using CO_2_ asphyxiation in accordance with Yale University IACUC protocol. The temporal bones were isolated and placed in 4% formaldehyde overnight after opening the round and oval windows. Cochlea were removed after micro-dissection of the temporal bones washed in incubation buffer three times and processed for immunostaining as described above.

### Cell transfection and imaging

MDCK and LLC-PK cells were a gift from Dr Michael Caplans lab, Yale University (in turn obtained from ATCC) and cells were not subject to STR profiling. MDCK and LLC-PK cells plated on glass coverslips (or where applicable HEK cells and CHO cells) were transiently transfected with constructs using Fugene6 according to the manufacturer's instructions (Promega, Madison, WI). Cells were plated at 100% confluency to ensure polarization, and transfected 36 hours after plating. Cells were fixed at 30 hours after transfection unless otherwise indicated. Cells were imaged by confocal microscopy using a Zeiss LSM 510/510 meta after fixation as previously described (Bai et al., 2011). Image parameters (scan time per pixel, pixel density, z step) were kept constant in a given experimental paradigm to allow reliable comparison.

In siRNA experiments, MDCK cells were electroporated with Prestin YFP and siRNA to the medium subunit of AP1B (μ1B) as previously described (Gravotta et al., 2007). We used a previously proven siRNA to μ1B siRNA (μ1B-M16 sequence 5′-AACAAGCTGGTGACTGGCAAA-3′ (Gravotta et al., 2007)] and a control chicken β-4 siRNA (chick KCNMB4, (Bai et al., 2011)) that were custom synthesized (Dharmacon, Lafayette, CO). Briefly, μ1B siRNA (or chick KCNMB4 siRNA in control experiments) at 5 nM and PrestinYFP plasmid at 10 nM was resuspended with 1 million trypsinized MDCK cells in 100 µl of DMEM. Cells were electroporated using a square wave pulse with the following parameters: 300 V for 100 µs×1 and 25 V for 20 ms×11 with a 100 ms pause in between. The entire electroporation protocol was repeated once. Cells were incubated on ice for 30 minutes and plated in prewarmed complete medium in 24 well plates on glass coverslips at a density of 350,000 cells per well (100% confluency). The medium was changed daily and cells were fixed and imaged 72 hours later.

Sixteen bit Images were acquired on a Zeiss 510 laser scanning microscope using a 63× water immersion lens (N.A 1.4), with fixed laser settings, a scan rate of 6.4 µs per pixel, a pinhole aperture of 1.0 Airy units, and fixed detector gain. Regions of interest were identified and fluorescence data extracted. We established that the fluorescence intensity was within the linear range and used mean fluorescence density as a measure of protein concentration.

### Gated STED

Gated STED, a new method of super resolution confocal microsopy, was used to visualize prestin expression in OHCs. Mouse cochlea were dissected and incubated with anti-prestin antibody (1:100 goat anti-prestin, Santa Cruz, CA) in incubation buffer overnight at 4°C. Tissue was washed three times with incubation buffer and then incubated with donkey anti-goat Oregon Green 488 (1:100, Beckton Dickinson) at room temperature for 1 hour. Cells were washed three times in incubation buffer followed by two washes in PBS. Tissue was mounted in Prolong Gold (Invitrogen) and viewed using a Leica TCS SP8 Gated STED microscope (Vicidomini et al., 2011).

### Image processing and data analysis

Images were processed after acquisition using Volocity software (Perkin Elmer, CA). Pearson's correlation was calculated after automated thresholding using the method of Costes et al. (Costes et al., 2004). All results are given as mean ± s.e.m. Where appropriate, ANOVA with Bonferroni Multiple Comparisons test was used to test for significance in differences (Instat3, CA).

### Electrophysiological recording

For electrophysiological recordings 100,000 Chinese hamster ovary (CHO) cells were transfected in 24-well plates using Lipofectamine (Invitrogen, Carlsbad, CA) as previously described (Bai et al., 2011).

Cells were recorded by whole-cell patch clamp configuration at room temperature using an Axon 200B amplifier (Axon Instruments, CA), as described previously (Bai et al., 2011). Cells were recorded 24–48 hours after transfection to allow for stable measurement of non-linear capacitance. Ionic blocking solutions were used to isolate capacitive currents. The bath solution contained (in mM): TEA 20, CsCl 20, CoCl_2_ 2, MgCl_2_ 1.47, Hepes 10, NaCl 99.2, CaCl_2_·2H_2_O 2, pH 7.2, and the pipette solution contained (in mM): CsCl 140, EGTA 10, MgCl_2_ 2, Hepes 10, pH 7.2. Osmolarity was adjusted to 300±2 mOsm with dextrose. Command delivery and data collections were carried out with a Windows-based whole-cell voltage clamp program, jClamp (Scisoft, New Haven, CT), using a Digidata 1322A interface (Axon Instruments, CA).

Capacitance was evaluated using a continuous high-resolution 2-sine wave. Capacitance data were fitted to the first derivative of a two-state Boltzmann function:
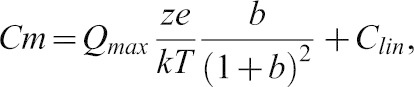
where
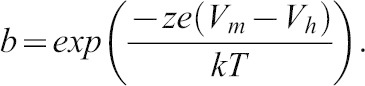
Q_max_ is the maximum nonlinear charge transfer, V_h_ the voltage at peak capacitance or half-maximal nonlinear charge transfer, V_m_ the membrane potential, C_lin_ linear capacitance, z the valence (a metric of voltage sensitivity), e the electron charge, k the Boltzmann's constant and T the absolute temperature. Q_max_ is reported as Q_sp_ the specific charge density, i.e. total charge moved normalized to linear capacitance. A students *t*-test was used to evaluate the effects of mutants on the different parameters of NLC.

## RESULTS

### OHCs express prestin and other basolateral markers along baso-lateral wall, and express the AP1B (µ1B) subunit

**Fig. 1.**
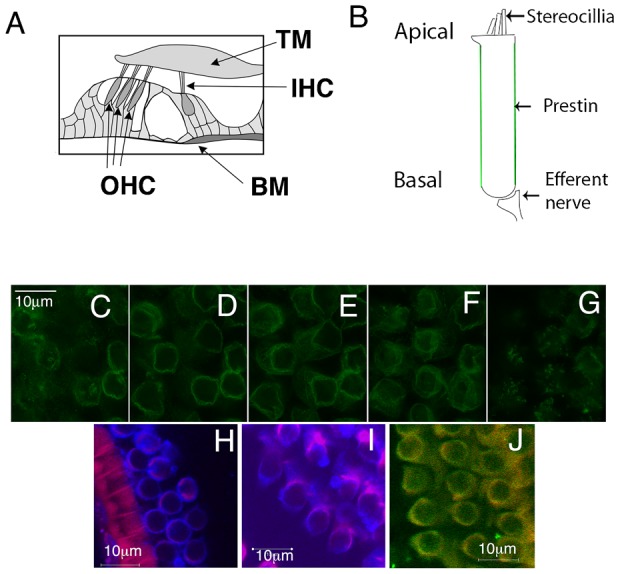
Prestin in mouse outer hair cells is localized along the lateral wall of the cell along with β-catenin and Na/K ATPase. Shown are cartoons of the organ of Corti (A) and its contained outer hair cells. TM, tectorial membrane; BM Basilar membrane; IHC, inner hair cell; OHC Outer hair cell. (B) A model of an outer hair cell in which prestin is shown lining its lateral wall (green). (C–G) The figure shows serial X-Y sections of mouse outer hair cells labeled with an anti-prestin antibody and then visualized using a Leica gated STED microscope. The sections start at the apical end (left) and end at the basal end (right). There is an uniform labeling of prestin along the lateral wall of the cell (middle three panels). Prestin labeling at the apical end of the cell tapers (C). Similarly, there is a patchy clustering of prestin at the basal pole of the cell (G). Mouse outer hair cells immunostained with antibodies to the basolateral markers β-catenin (H) and Na/K ATPase (I), and demonstrates the localization of these proteins (blue) along the lateral wall of the cell. The cells were counterstained with phalloidin Alexa 546 (red), which shows the presence of the sub cortical lattice of actin along the lateral wall of the cell. (J) The AP1µ1B subunit (green) is present in outer hair cells evidenced by antibody labeling of these cells. The figure shows co labeling of these cells with Na/K ATPase (red). Scale bar is 10 microns. These experiments were repeated five times.

Previous theoretical treatments confirmed by electrophysiological and immunolocalization experiments have demonstrated the localization of prestin along the lateral wall of OHCs. We sought to further confirm these data using high resolution confocal microscopy. Adult mice cochlea were stained with antiprestin antibodies and visualized using gated STED microscopy. As shown in Fig. 1 prestin is sharply localized along the lateral wall of the OHCs. The protein staining was uniform through the lateral wall. At the basolateral pole of the cell however, there was a patchy clustering of prestin. In contrast, there was a gradual tapering of prestin staining at the apical end of the cell.

The localization of prestin to the basolateral surface of outer hair cells led us to reason that outer hair cells are analogous to polarized epithelial cells with anatomical and functional segregation of the cell into an apical and basolateral compartments. The apical segregation of the stereociliary apparatus and the basolateral segregation of the cochlear amplifier are consistent with this separation. Prior work in hair cells of different species has shown the presence of β-catenin and Na/K ATPase, two proteins identified as classically localized in the basolateral compartment of polarized epithelial cells, to be present in the basolateral surface of hair cells. As shown in Fig. 1 these two proteins – β-catenin and Na/K ATPase – were found to segregate along the basolateral surface of outer hair cells (Fig. 1), further confirming similarities between outer hair cells and polarized epithelial cells. We also determine that OHCs express the AP1B (μ1β) subunit that has been shown to be important for basolateral sorting of proteins in polarized epithelial cells (Fig. 1).

### Prestin has several tyrosine residues within a basolateral targeting sequence of which Y520 and Y667 are important for basolateral sorting

**Fig. 2.**
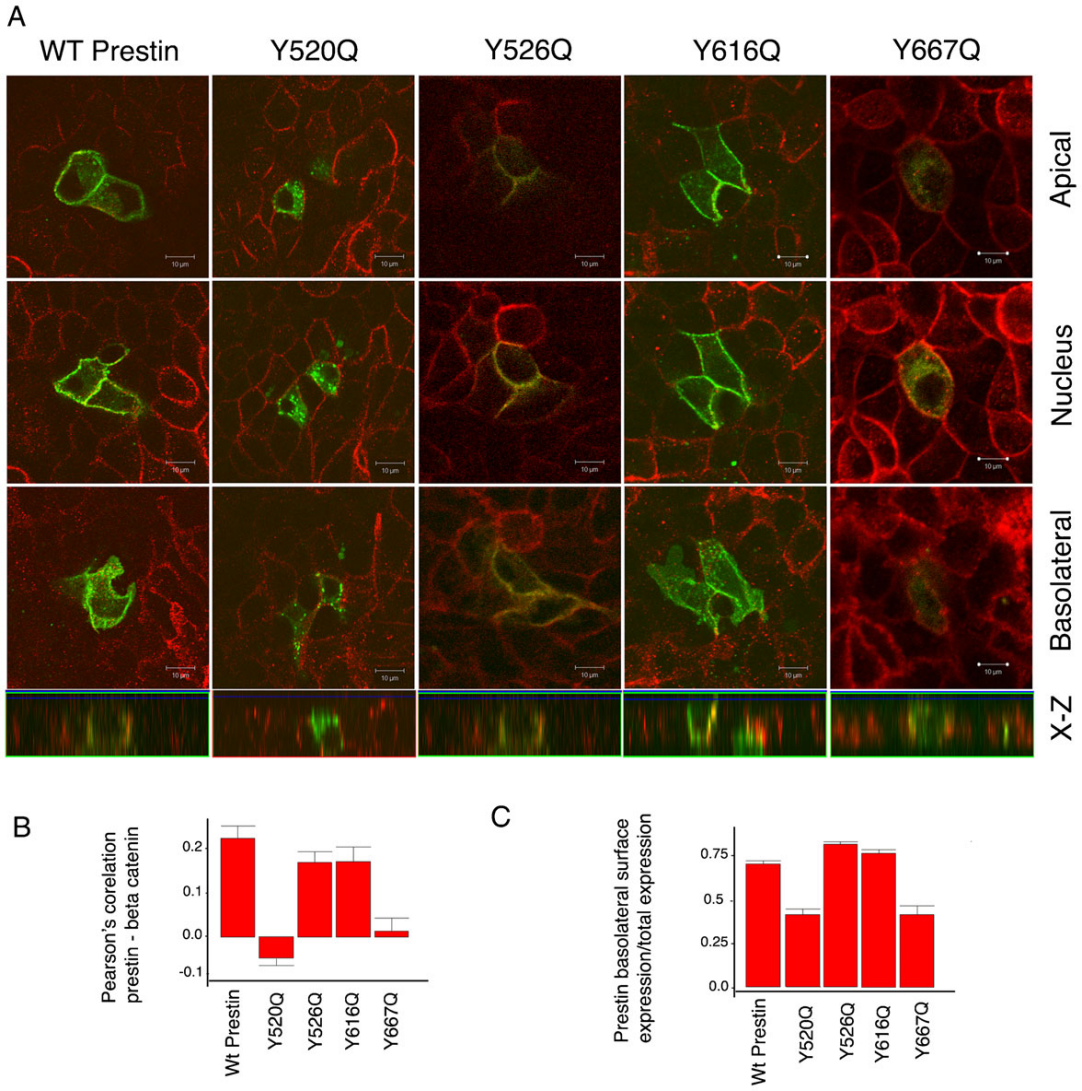
Y520 and Y667 contained within the YXXΦ motif are important for basolateral targeting of prestin in MDCK cells. The figure shows MDCK cells transiently transfected with wild type prestin and mutations of prestin: Y520Q, Y526Q, Y616Q and Y667Q. The cells were fixed after 36 hours. Prestin was tagged with YFP at its C-terminus (green) and the cells were stained with an antibody to β-catenin. Shown are serial X-Y confocal sections along the z axis (A). The corresponding X-Z sections are shown at the bottom. Mutations at Y520 and Y667 result in a failure to target the basolateral surface of the cell along with intracellular retention of the protein and apical trafficking. (B,C) Pearson's correlation of prestin with β-catenin and a ratio of prestin fluorescence on the surface of the cell compared to the total in the cell. The mean Pearson's correlation values were: wt 0.23 (+/−0.028SE, *n* = 7); Y520Q −0.045 (+/−0.016 SE, *n* = 7); Y526Q 0.17 (+/−0.025 SE, *n* = 14); Y616Q 0.175 (+/−0.033 SE, *n* = 14); Y667Q 0.01386A (+/−.03 SE, *n* = 10). The differences between wt and Y520Q and Y667Q were considered significant. A one way ANOVA (parametric) with Bonferroni post test comparison yielded a p value of <0.001 for wt vs Y520Q and wt vs Y667Q; wt vs Y616Q and Y667Q were not significant. The mean surface to total ratios were: wt 0.67 (+/−0.016 SE, *n* = 22); Y520Q −0.39 (+/−0.0129 SE, *n* = 20); Y526Q 0.78 (+/−0.013 SE, *n* = 13); Y616Q 0.73 (+/−0.02 SE, *n* = 13); Y667Q 0.40 (+/−.044 SE, *n* = 13). Here too the differences between wt and Y520Q and Y667Q were significant. A one-way ANOVA (parametric) with Bonferroni post-test comparison yielded a p value of <0.001 for wt vs Y520Q and wt vs Y667Q. The scale bar is 10 microns.

In order to establish an experimental model system to tease apart the mechanisms underlying prestins basolateral targeting, we expressed it in MDCK cells. These cells have a long history of being used as a model system for studying polarized sorting in epithelial cells. As shown in Fig. 2 prestin tagged with YFP at its C-terminus is targeted to the basolateral surface of MDCK cells. The protein shows a similar pattern of distribution to β-catenin and Na/K ATPase, both well known basolaterally targeted proteins. Prestin lacking a YFP tag showed similar targeting to the basolateral membrane (data not shown).

**Fig. 3.**
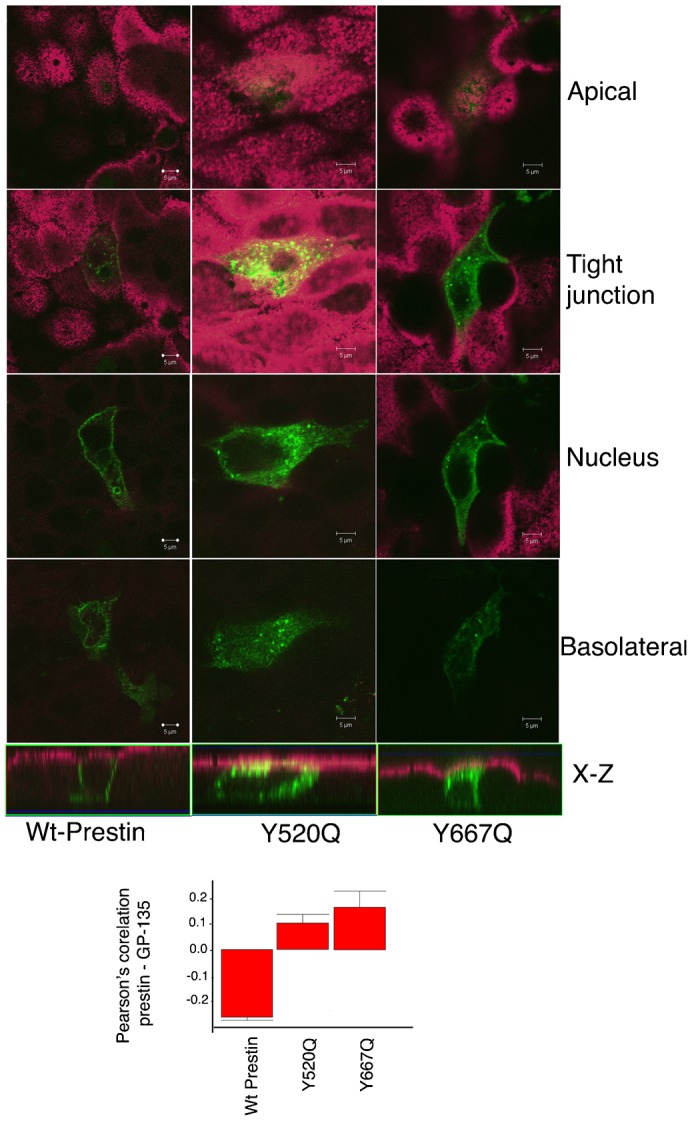
Mutation of Y520 and Y667 result in increased delivery of prestin to the apical surface of MDCK cells. MDCK cells transiently transfected with wt prestin YFP and the two constructs Y520Q prestin YFP, and Y667Q prestin YFP were fixed at 36 hours and stained with antibody to the apical marker GP130 (podohexin). There is significant apical targeting of Y520Q and Y667Q evident in the serial X-Y sections along the z axis and the corresponding X-Z sections at the bottom. The bottom panel shows Pearson's correlation of prestin YFP and GP130 confirming absent apical targeting of the wild type construct and apical targeting of Y520Q and Y667Q. The mean Pearson's correlation values were: wt −0.027 (+/−0.011 SE, *n* = 7); Y520Q 0.105 (+/−0.035 SE, *n* = 5); Y667Q 0.17 (+/−0.063 SE, *n* = 11). The differences between wt prestin and Y520Q and wt prestin and Y667Q were significant. A one-way ANOVA (parametric) yielded a p value of <0.01 between wt prestin and Y520Q, and a p value of <0.001 between wt prestin and Y667Q. The scale bar is 5 microns.

A number of sequence motifs in a protein are important for the targeting of a protein to the basolateral surface of polarized epithelial cells. Prestin has several tyrosine residues contained within a YXXΦ motif in its C-terminus, and lacks other targeting motifs classically associated with basolateral targeting (including dileucine motifs, NPxY) (Brewer and Roth, 1991; Cancino et al., 2007; Gonzalez and Rodriguez-Boulan, 2009; Gravotta et al., 2007; Hunziker et al., 1991; Lin et al., 1997; Matter et al., 1992; Rodriguez-Boulan and Gonzalez, 1999; Weisz and Rodriguez-Boulan, 2009). These include the tyrosine residues Y520, Y526, Y616 and Y667 (shown in cartoon form in the two alternative membrane spanning models of prestin in supplementary material Fig. S1). In order to ascertain the importance of these residues for basolateral targeting, we individually mutated these residues and determined their basolateral localization. As shown in Fig. 2 mutation of Y520Q, and to a lesser extent Y667Q, resulted in a reduced expression of prestin on the basolateral surface of the cell. There was both increased intracellular retention (Fig. 2) of the protein as well as targeting to the apical surface of the cell (Fig. 3).

**Fig. 4.**
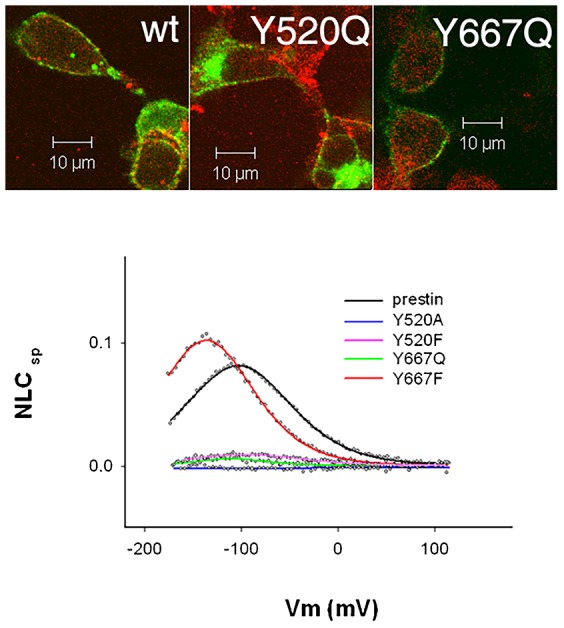
Mutation of Y520Q and Y667Q results in targeting of prestin to the plasma membrane of HEK cells, and presence of NLC in Y667Q, Y520F and Y667F. The upper panels show HEK cells transfected with wild type prestin-YFP, and the two constructs Y520Q prestin YFP, and Y667Q prestin YFP that were fixed 48 hours after transfection. Cells were stained with antibodies to Na/K ATPase and visualized by confocal microscopy. Wild type prestin YFP, Y520Q prestin YFP and Y667Q prestin YFP all target the plasma membrane as evidenced by its co-localization with plasma membrane Na/K ATPase. The lower panel shows NLC traces of different mutations at Y520 and Y667. The actual values of NLC parameters along with cell numbers are given in supplementary material Table S1. The scale bar is 10 microns.

In order to further confirm that the effects of these mutations were specific in bringing about deficient basolateral targeting we sought to determine the effects of these mutations on the surface expression of prestin in non-polarized HEK and CHO cells. As shown in Fig. 4 wt type prestin and prestin with mutations at Y520 and Y667 were both targeted to the cell surface. However, both these mutations affected the function of the molecule. In CHO cells expressing these mutant proteins, an increase in non-linear capacitance, the widely accepted functional surrogate for electromotility, was absent with Y520Q and markedly diminished with Y667Q. Supplementary material Table S1 shows the parameters for NLC in these mutants.

### The phenol ring within the tyrosine residues (Y520 and Y667) are important for basolateral targeting

**Fig. 5.**
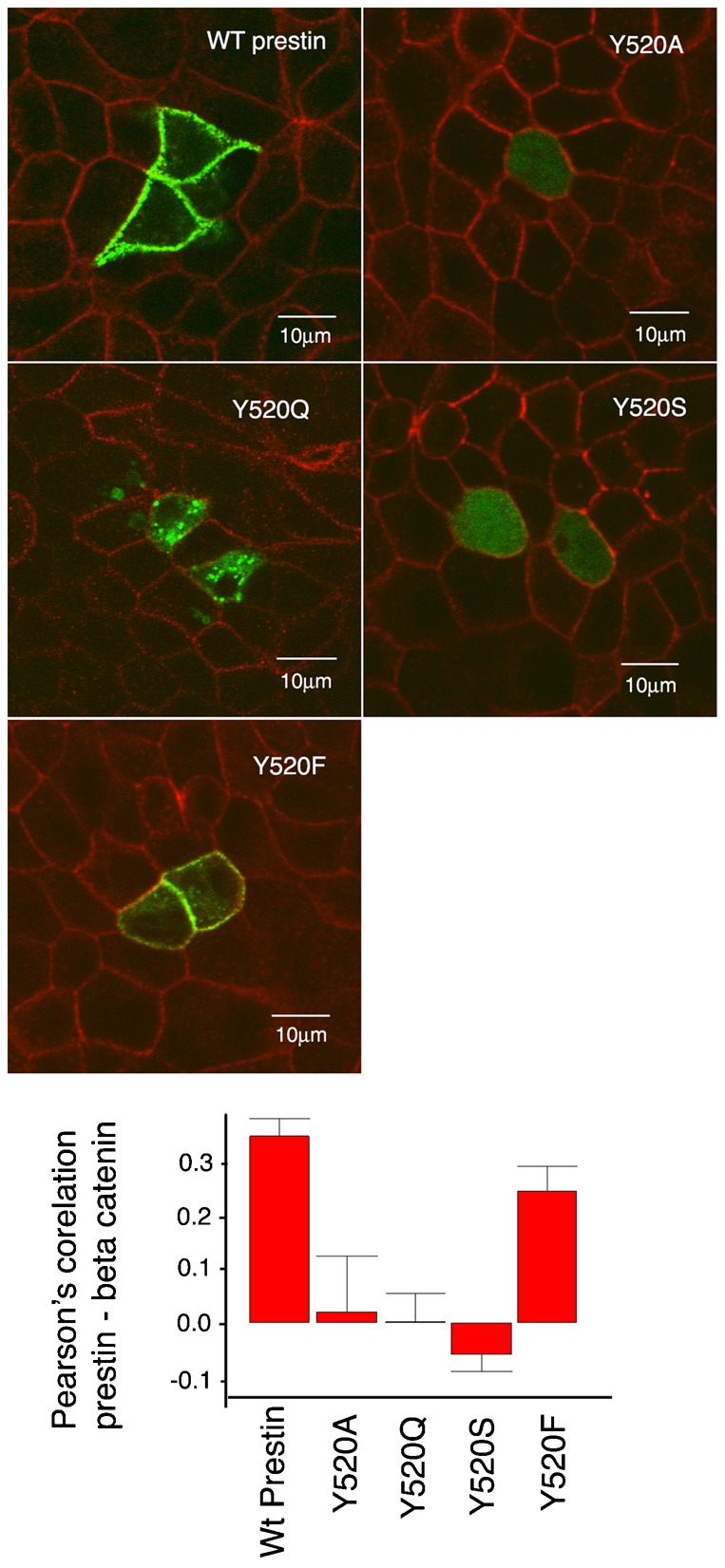
The phenol ring in the tyrosine residue is critical for the targeting of prestin to the basolateral surface of MDCK cells. MDCK cells transiently transfected with prestin YFP and the mutations Y520Q, Y520A, Y520S and Y520F were fixed 36 hours after transfection and imaged by confocal microscopy. The basolateral wall of the cell was visualized by immunostaining with anti-β-catenin antibody. The mutations Y520A, Y520Q, and Y520S failed to target the basolateral surface of the cell, while Y520F was targeted to the basolateral surface of the cell. Pearson's correlation between prestin YFP and β–catenin confirm the observed co-localization of wild type prestin-YFP with β-catenin and Y520F with β-catenin. The mean Pearson's correlation values were: wt prestin 0.34 (+/−0.033 SE, *n* = 8); Y520A 0.019 (+/−0.10 SE, *n* = 5); Y520Q 0.002 (+/−0.052 SE, *n* = 5); Y520S −0.05 (+/−0.03, *n* = 6); Y520F 0.25 (+/−0.045 SE, *n* = 6). A one way ANOVA revealed significant differences between wt prestin and Y520A (*P*<0.01), wt prestin and Y520Q (*P*<0.001), and wt prestin and Y520S (*P*<0.001). The differences between wt prestin and Y520F were not significant. The scale bar is 10 microns.

We sought to determine the importance of the stereo chemical properties of the tyrosine residue at position 520 and 667 to ascertain its effects on targeting to the basolateral surface of MDCK cells. We mutated individual tyrosine residues to serine or phenylalanine to mimic the effects of the side chain hydroxyl group and phenol ring respectively. We determined that substitution of tyrosine with phenylalanine at Y520 resulted in near complete recovery in basolateral targeting (Fig. 5). In contrast, mutation of Y520 to a serine residue (to mimic the side chain OH group) resulted in poor delivery of the protein to the basolateral surface (Fig. 5). Similarly, it is unlikely that the effects of these other mutations are mediated by their size or hydrophilicity since mutation of Y520 to alanine failed to restore basolateral targeting of prestin (Fig. 5). Similar effects were seen with mutation of Y667.

### Mutation of Y520 and Y667 residues results in retention within the Golgi

**Fig. 6.**
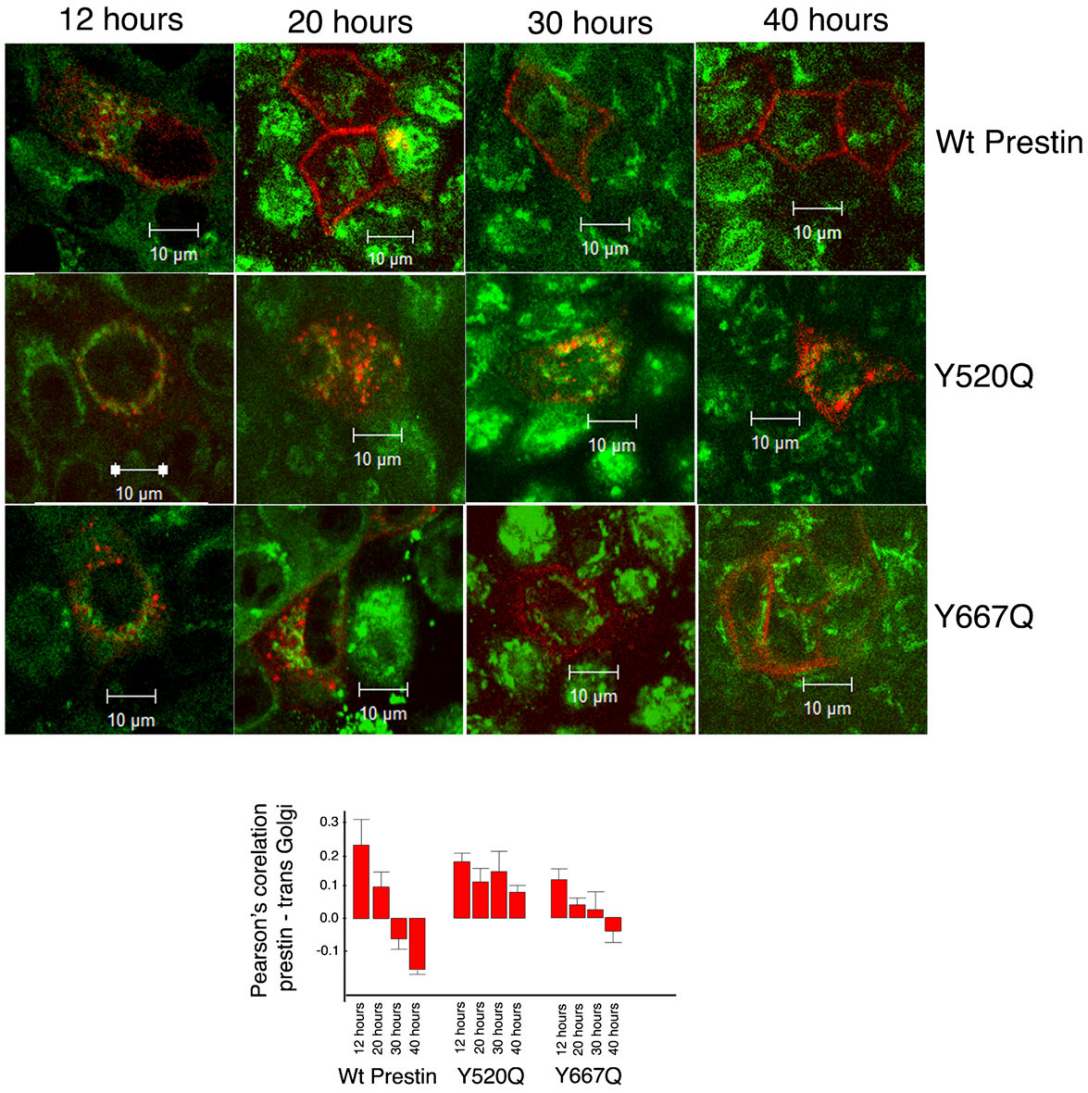
Egress from the Golgi is poor/absent in Y520Q and delayed in Y667Q. MDCK cells transiently transfected with prestin YFP, and the mutations Y520Q and Y667Q contained in the prestin YFP cassette were fixed after 12, 20, 30 and 40 hours after transfection. The cells were then stained with antibodies to the Golgi protein Giantin. While prestin YFP exits the Golgi at 20 hours, Y520 dos not egress from the Golgi and Y667Q leaves the Golgi after 40 hours. A graphical form of Pearson's correlation between prestin YFP and Giantin at the different time points is shown below. For wt prestin there was a significant difference on one way ANOVA between the Pearson's correlation between prestin and Giantin at all times compared to 12 hours: 12 hours vs 20 hours, *P*<0.05; 12 hours vs 30 hours, *P*<0.001; and 12 hours vs 40 hours *P*<0.001. In contrast, the differences in Pearson's correlation between Y520Q and Giantin at all times compared to 12 hours were not significantly different (*P*>0.05 on one way ANOVA). For Y667Q the Pearson's correlation with Giantin was significantly different on one way ANOVA between 12 hours and 40 hours (*P*<0.05), while the remaining two time points were not significantly different (12 hours vs 20 hours, *P*>0.05; 12 hours vs 30 hours, *P*>0.05). The mean values for the different time points were as follows: wt prestin 12, 20, 30 and 40 hours: 0.23 (+/−0.08 SE, *n* = 5), 0.09 (+/−0.04 SE, *n* = 10), −0.06 (+/−0.03 SE, *n* = 12), −0.162 (+/−0.02 SE, *n* = 13); Y520Q 12, 20, 30 and 40 hours: 0.17 (+/−0.02 SE, *n* = 5), 0.114 (+/−0.04 SE, *n* = 6), 0.145 (+/−0.06 SE, *n* = 13), 0.08 (+/−0.02 SE, *n* = 8); Y667Q 12, 20, 30 and 40 hours: 0.112 (0.03 SE, *n* = 13), 0.04 (+/−0.02 SE, *n* = 9); 0.026 (+/−0.05 SE, *n* = 8) and −0.04(+/−0.03 SE, *n* = 8). The scale bar is 10 microns.

Previous experiments in other proteins targeted to the basolateral surface have shown that sorting of proteins is determined at the Golgi. We hypothesized that mutation of tyrosine residues at Y520 and Y667 would result in retention of the protein within the Golgi (and could explain at least partially, the increased amounts of the mutated protein retained within the cell). MDCK cells transfected with prestin YFP, Y520Q, or Y667Q were fixed at different times after transfection and retention within the Golgi determined by co-localization with the Golgi specific protein Giantin (Fig. 6) (Deborde et al., 2008). We determined that mutation of Y520Q resulted in a persistent retention of prestin within the Golgi. In contrast, Y667Q showed retention of prestin within the Golgi, which improved with time. Thus, Y667Q was localized within the Golgi at 12, 20 and 30 hours after transfection but demonstrated increasing efflux from the Golgi at 40 hours. In contrast, wt prestin had made a near complete exit from the Golgi by 20 hours. Y520Q showed little egress from the Golgi with continued retention within that organelle even at 40 hours.

### Prestin exit from the Golgi to the plasma membrane is mediated by early recycling endosomes

**Fig. 7.**
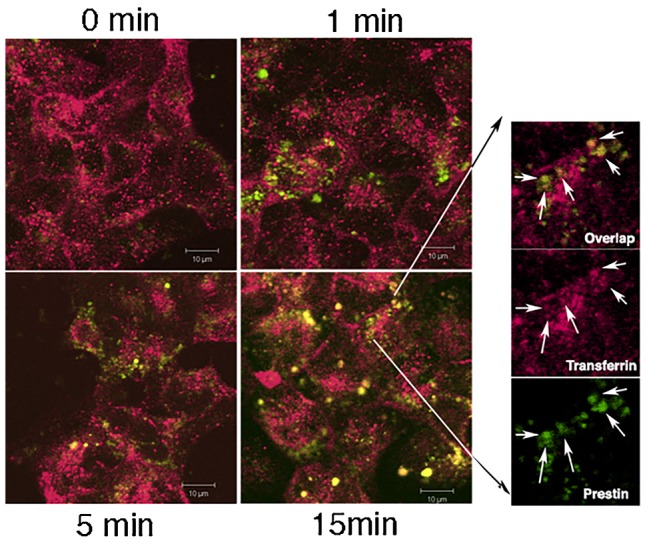
Prestin YFP uses transferrin containing endosomes to transit from the Golgi to the basolateral membrane. MDCK cells were transiently transfected with prestin-YFP and kept at 19°C to induce Golgi block. Cells were incubated with transferrin - Alexa 647 for 10 minutes. The incubation temperature was raised to 37°C after which cells were fixed at 0, 1 minute, 5 minutes, 10 minutes and 15 minutes. Cells were then imaged with confocal microscopy. Shown are confocal images in the X-Y plane at the basolateral Z axis of the cell that were fixed at different times after raising the temperature to 37°C. With increasing time there is co-localization of prestin-YFP exiting the Golgi with transferring - Alexa 647. The right hand panels shows merged and individual Alexa 647 and prestin-YFP images of an enlarged area at 15 minutes after raising the temperature to 37°C. Co-localization of prestin YFP and transferrin 647 is demonstrated. The scale bar is 10 microns.

We then sought to determine the identity of organelles responsible for movement of prestin from the Golgi to the plasma membrane. For these experiments we transfected prestin YFP into MDCK cells. Transfected cells were kept at 19°C to allow accumulation of protein within the Golgi (Golgi block) (Bian et al., 2013). Cells were incubated with transferrin Alexa 647 applied to cells for 10 minutes to label early/recycling endosomes. The cells were then immersed in fresh culture medium and fixed at different time points after raising the temperature to 37°C to release proteins from Golgi block. As shown in Fig. 7 there was co-localization of vesicles containing both prestin YFP and transferrin Alexa 647. We interpret this data to suggest that prestin YFP use early/recycling endosomes to reach the basolateral surface of the cell.

### Prestin targeting to the basolateral surface also involves the AP1B (μ1B) pathway

**Fig. 8.**
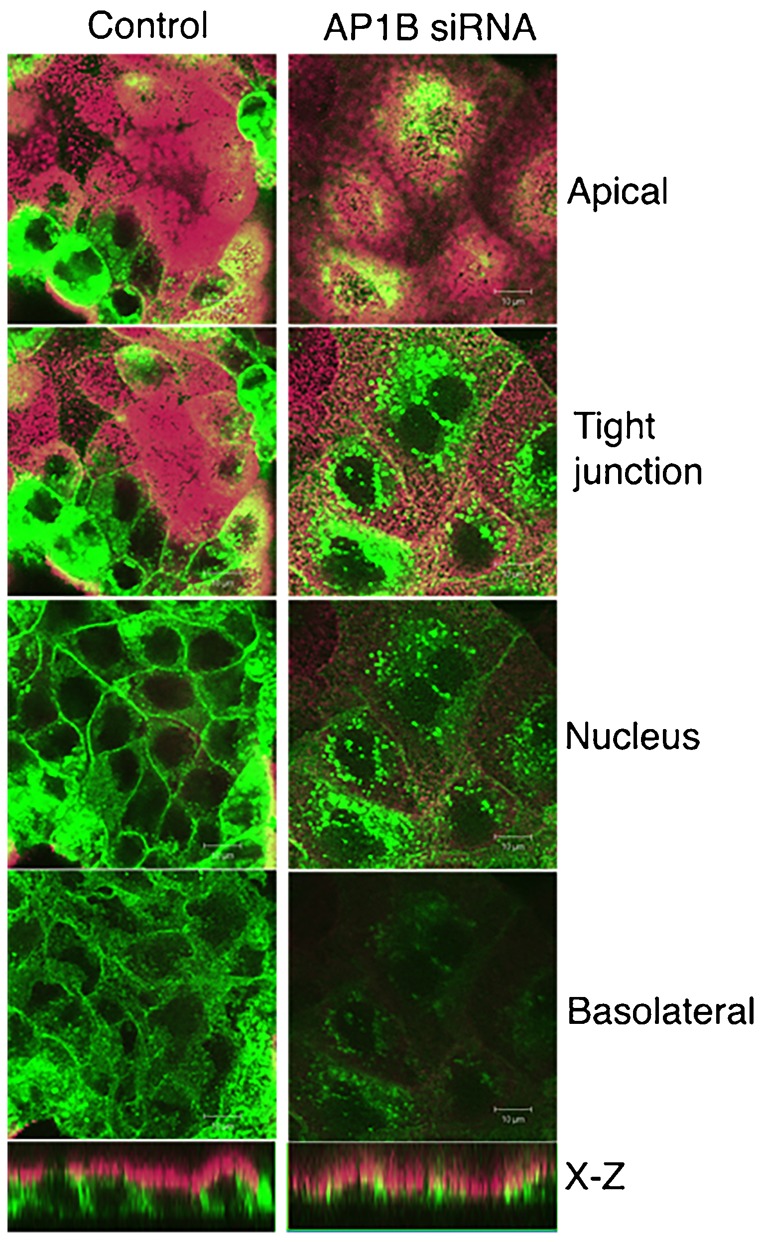
Targeting of prestin YFP to the basolateral membrane requires AP1B (μ1B). MDCK cells were electroporated with prestin YFP plasmid and siRNA to AP1B (μ1B). Cells were plated at confluent density and fixed after 30 hours. Cells were stained with antibodies to the apical marker GP130 and visualized by confocal microscopy. Shown are X-Y images along the z axis of transfected cells. The lowest panel shows the corresponding X-Z sections. Transfection of MDCK cells with siRNA to AP1B (μ1B) resulted in an apical localization of prestin YFP and near absence in targeting to the basolateral surface. In contrast, cells co-transfected with control siRNA (chicken KCNMB4) resulted in basolateral targeting of prestin YFP. The scale bar is 10 microns.

In other systems the use of tyrosine motifs for basolateral sorting in polarized epithelial cells has been shown to involve the AP1B (μ1B) pathway. We sought to ascertain if AP1B (μ1B) was important for sorting prestin to the basolateral surface of polarized epithelial cells. In initial experiments we used LLC-PK cells, which lack the AP1B (μ1B) subunit. In these cells Prestin YFP shows decreased targeting to the basolateral surface of the cell (supplementary material Fig. S2). We then used siRNA knockdown of AP1B (μ1B) to determine targeting of prestin YFP in MDCK cells. In these cells knock down treatment with siRNA to AP1B (μ1B) resulted in apical targeting of the protein and reduced expression of the protein on the basolateral surface of the cell (Fig. 8).

## DISCUSSION

In this paper we show for the first time that prestin is sorted to the basolateral surface of polarized epithelial (MDCK) cells using a tyrosine motif. We identified two tyrosine residues contained in the cassette YXXΦ motif that are likely important for its localization to the basolateral surface of the cell. Mutation of one of these residues Y520 resulted in apical targeting and intracellular retention of the protein. A second mutation of Y667 resulted in delayed exit from the Golgi to the basolateral surface with initial targeting to the apical surface of the cell and intracellular retention. With increasing time, however, there was progressive localization of this mutant at the basolateral surface of the cell. We believe mutation of these residues resulted in specific loss of basolateral targeting since the mutant protein was targeted to the apical surface of MDCK cells and since the protein showed plasma membrane targeting in non-polarized CHO and HEK cells. Nevertheless, it still remains a possibility that the loss of basolateral targeting resulted from a subtle misfolding of the protein that also affected its normal function. We were unable to demonstrate normal function as measured by the presence of non-linear capacitance in these mutants expressed in CHO cells (Y520Q showed absent NLC while Y667Q showed diminished NLC). In this context, it should be noted that several mutations that also affected prestin function by subtle alterations in its folding, show normal basolateral targeting in MDCK cells (data not shown). We would also like to note previous experiments by Zheng et al. (Zheng et al., 2005), in which mutation of Y520 and Y526 together failed to show targeting to the surface of TSA and OK cells. We do not have an explanation for the discordance in our data although the experiments are not strictly comparable since Zheng et al. (Zheng et al., 2005) were describing the targeting of a double mutant (Y520A and Y526A) while we describe the targeting of single mutations (Y520 or Y667).

Our interpretation that prestin is sorted to the basolateral surface of cells using a tyrosine motif is further substantiated by two other related findings. First, we demonstrate that AP1B (μ1B) is important for basolateral targeting, since expression of prestin in LLC-PK cells that lack this subunit resulted in decreased basolateral targeting. Moreover knockdown of AP1B (μ1B) in MDCK cells also resulted in increased apical targeting of prestin with deficient basolateral targeting of the protein. It is now believed that the tyrosine residues are critical for direct interaction with AP1B (μ1B) and bringing about basolateral targeting of proteins (Bonifacino and Dell'Angelica, 1999; Carvajal-Gonzalez et al., 2012; Fields et al., 2007). We also demonstrate using Golgi block experiments that prestin uses recycling endosomes as a post Golgi transport intermediate to transit from the Golgi to the basolateral plasma membrane. The use of AP1B (μ1B) has been demonstrated by several other proteins using tyrosine residues contained in a YXXΦ motif to target the basolateral membrane (Adair-Kirk et al., 2003; Ang et al., 2004; Duffield et al., 2004; Fields et al., 2007; Fölsch et al., 1999; Fölsch et al., 2003; Gravotta et al., 2007; Sugimoto et al., 2002). Moreover, the use of transferrin containing endosomes has been shown with basolateral proteins that use a tyrosine motif to exit the Golgi (Ang et al., 2004; Donoso et al., 2009). In prior experiments membrane targeting to the surface of CL4 cells, which are derived from LLC-PK1 cells have been observed (Zheng et al., 2010). In the absence of data on the expression of AP1B (μ1B) in CL4 cells used for these experiments or the relative surface expression of prestin in these cells, it is difficult to comment on the significance of these data (Zheng et al., 2010).

The implications to our data are greatest to hair cells. Our data further suggest that hair cells are similar to polarized epithelial cells with differential sorting of proteins to the apical and basolateral surface of the cell. Hair cells could be thought of as having different compartments – an apical surface important for transducing sound and a basal pole that is important for synaptic transmission in inner hair cells and a basolateral pole that is important for housing the cochlear amplifier and its processes. Our data suggest that integral to this segregation of function is the segregation of proteins to different apical and basal compartments of the cell along the lines demonstrated in polarized epithelial cells. Other work has also shown the apical sorting in CL4 cells of proteins identified as important for stereociliary and transduction function in hair cells (Zheng et al., 2010). At least in the context of protein sorting, hair cells seem to have retained features of polarized epithelial cells rather than neurons. The expected segregation of proteins along a dendritic and axonal separation would result in a reversal of the observed localization of proteins in hair cells. Perhaps one explanation for this unexpected finding is that hair cells resemble epithelial cells more than they do neurons. Importantly, hair cells are derived from the embryonic otic placode, a thickened segment of the cranial ectoderm that invaginates separately from the neural tube to form the otocyst. Consistent with its epithelial phenotype, hair cells express the clathrin adaptor protein AP1B (μ1B) subunit that is present only in polarized epithelial cells and lacking in neurons. Significantly, this subunit is important for the localization of several proteins that, like prestin, use a tyrosine motif to target to the basolateral surface of the cell. It remains to be established if prestin uses tyrosine residues and /or the AP1B (μ1B) subunit to target to the lateral wall of outer hair cells.

## Supplementary Material

Supplementary Material

## References

[b1] Adair-Kirk T. L. Dorsey F. C. Cox J. V. (2003). Multiple cytoplasmic signals direct the intracellular trafficking of chicken kidney AE1 anion exchangers in MDCK cells. J. Cell Sci. 116, 655–663. 10.1242/jcs.00260 12538766

[b2] Ang A. L. Taguchi T. Francis S. Fölsch H. Murrells L. J. Pypaert M. Warren G. Mellman I. (2004). Recycling endosomes can serve as intermediates during transport from the Golgi to the plasma membrane of MDCK cells. J. Cell Biol. 167, 531–543. 10.1083/jcb.200408165 15534004 PMC2172492

[b3] Ashmore J. F. (1987). A fast motile response in guinea-pig outer hair cells: the cellular basis of the cochlear amplifier. J. Physiol. 388, 323–347. 3656195 10.1113/jphysiol.1987.sp016617 PMC1192551

[b4] Bai J. P. Surguchev A. Navaratnam D. (2011). β4-subunit increases Slo responsiveness to physiological Ca2+ concentrations and together with β1 reduces surface expression of Slo in hair cells. Am. J. Physiol. 300, C435–C446. 10.1152/ajpcell.00449.2010 PMC3063969 21178105

[b5] Bian S. Bai J. P. Chapin H. Le Moellic C. Dong H. Caplan M. Sigworth F. J. Navaratnam D. S. (2011). Interactions between β-catenin and the HSlo potassium channel regulates HSlo surface expression. PLoS ONE 6, e28264 10.1371/journal.pone.0028264 22194818 PMC3237428

[b6] Bian S. Navaratnam D. Santos-Sacchi J. (2013). Real time measures of prestin charge and fluorescence during plasma membrane trafficking reveal sub-tetrameric activity. PLoS ONE 8, e66078 10.1371/journal.pone.0066078 23762468 PMC3677934

[b7] Bonifacino J. S. Dell'Angelica E. C. (1999). Molecular bases for the recognition of tyrosine-based sorting signals. J. Cell Biol. 145, 923–926. 10.1083/jcb.145.5.923 10352010 PMC2133128

[b8] Bradke F. Dotti C. G. (1998). Membrane traffic in polarized neurons. Biochim. Biophys. Acta 1404, 245–258. 10.1016/S0167-4889(98)00060-3 9714822

[b9] Brewer C. B. Roth M. G. (1991). A single amino acid change in the cytoplasmic domain alters the polarized delivery of influenza virus hemagglutinin. J. Cell Biol. 114, 413–421. 10.1083/jcb.114.3.413 1860878 PMC2289095

[b10] Brownell W. E. Bader C. R. Bertrand D. de Ribaupierre Y. (1985). Evoked mechanical responses of isolated cochlear outer hair cells. Science 227, 194–196. 10.1126/science.3966153 3966153

[b11] Cancino J. Torrealba C. Soza A. Yuseff M. I. Gravotta D. Henklein P. Rodriguez-Boulan E. González A. (2007). Antibody to AP1B adaptor blocks biosynthetic and recycling routes of basolateral proteins at recycling endosomes. Mol. Biol. Cell 18, 4872–4884. 10.1091/mbc.E07-06-0563 17881725 PMC2096610

[b12] Carvajal-Gonzalez J. M. Gravotta D. Mattera R. Diaz F. Perez Bay A. Roman A. C. Schreiner R. P. Thuenauer R. Bonifacino J. S. Rodriguez-Boulan E. (2012). Basolateral sorting of the coxsackie and adenovirus receptor through interaction of a canonical YXXΦ motif with the clathrin adaptors AP-1A and AP-1B. Proc. Natl. Acad. Sci. USA 109, 3820–3825. 10.1073/pnas.1117949109 22343291 PMC3309744

[b13] Clemens Grisham R. Kindt K. Finger-Baier K. Schmid B. Nicolson T. (2013). Mutations in ap1b1 cause mistargeting of the Na(+)/K(+)-ATPase pump in sensory hair cells. PLoS ONE 8, e60866 10.1371/journal.pone.0060866 23593334 PMC3625210

[b14] Costes S. V. Daelemans D. Cho E. H. Dobbin Z. Pavlakis G. Lockett S. (2004). Automatic and quantitative measurement of protein-protein colocalization in live cells. Biophys. J. 86, 3993–4003. 10.1529/biophysj.103.038422 15189895 PMC1304300

[b15] Dallos P. Evans B. N. (1995). High-frequency motility of outer hair cells and the cochlear amplifier. Science 267, 2006–2009. 10.1126/science.7701325 7701325

[b16] Dallos P. Evans B. N. Hallworth R. (1991). Nature of the motor element in electrokinetic shape changes of cochlear outer hair cells. Nature 350, 155–157. 10.1038/350155a0 2005965

[b17] Davis H. (1983). An active process in cochlear mechanics. Hear. Res. 9, 79–90. 10.1016/0378-5955(83)90136-3 6826470

[b18] Deborde S. Perret E. Gravotta D. Deora A. Salvarezza S. Schreiner R. Rodriguez-Boulan E. (2008). Clathrin is a key regulator of basolateral polarity. Nature 452, 719–723. 10.1038/nature06828 18401403 PMC4078870

[b19] Donoso M. Cancino J. Lee J. van Kerkhof P. Retamal C. Bu G. Gonzalez A. Cáceres A. Marzolo M. P. (2009). Polarized traffic of LRP1 involves AP1B and SNX17 operating on Y-dependent sorting motifs in different pathways. Mol. Biol. Cell 20, 481–497. 10.1091/mbc.E08-08-0805 19005208 PMC2613102

[b20] Dotti C. G. Simons K. (1990). Polarized sorting of viral glycoproteins to the axon and dendrites of hippocampal neurons in culture. Cell 62, 63–72. 10.1016/0092-8674(90)90240-F 2163770

[b21] Dotti C. G. Parton R. G. Simons K. (1991). Polarized sorting of glypiated proteins in hippocampal neurons. Nature 349, 158–161. 10.1038/349158a0 1670898

[b22] Duffield A. Fölsch H. Mellman I. Caplan M. J. (2004). Sorting of H,K-ATPase beta-subunit in MDCK and LLC-PK cells is independent of mu 1B adaptin expression. Traffic 5, 449–461. 10.1111/j.1398-9219.2004.00192.x 15117319

[b23] Farr G. A. Hull M. Mellman I. Caplan M. J. (2009). Membrane proteins follow multiple pathways to the basolateral cell surface in polarized epithelial cells. J. Cell Biol. 186, 269–282. 10.1083/jcb.200901021 19620635 PMC2717640

[b24] Fettiplace R. (2009). Defining features of the hair cell mechanoelectrical transducer channel. Pflugers Arch. 458, 1115–1123. 10.1007/s00424-009-0683-x 19475417 PMC2745616

[b25] Fields I. C. Shteyn E. Pypaert M. Proux-Gillardeaux V. Kang R. S. Galli T. Fölsch H. (2007). v-SNARE cellubrevin is required for basolateral sorting of AP-1B-dependent cargo in polarized epithelial cells. J. Cell Biol. 177, 477–488. 10.1083/jcb.200610047 17485489 PMC2034334

[b26] Fölsch H. Ohno H. Bonifacino J. S. Mellman I. (1999). A novel clathrin adaptor complex mediates basolateral targeting in polarized epithelial cells. Cell 99, 189–198. 10.1016/S0092-8674(00)81650-5 10535737

[b27] Fölsch H. Pypaert M. Maday S. Pelletier L. Mellman I. (2003). The AP-1A and AP-1B clathrin adaptor complexes define biochemically and functionally distinct membrane domains. J. Cell Biol. 163, 351–362. 10.1083/jcb.200309020 14581457 PMC2173537

[b28] Gao J. Wang X. Wu X. Aguinaga S. Huynh K. Jia S. Matsuda K. Patel M. Zheng J. Cheatham M. (2007). Prestin-based outer hair cell electromotility in knockin mice does not appear to adjust the operating point of a cilia-based amplifier. Proc. Natl. Acad. Sci. USA 104, 12542–12547. 10.1073/pnas.0700356104 17640919 PMC1941505

[b29] Geisler C. D. (1993). A realizable cochlear model using feedback from motile outer hair cells. Hear. Res. 68, 253–262. 10.1016/0378-5955(93)90129-O 8407611

[b30] Geisler C. D. Sang C. (1995). A cochlear model using feed-forward outer-hair-cell forces. Hear. Res. 86, 132–146. 10.1016/0378-5955(95)00064-B 8567410

[b31] Glowatzki E. Grant L. Fuchs P. (2008). Hair cell afferent synapses. Curr. Opin. Neurobiol. 18, 389–395. 10.1016/j.conb.2008.09.006 18824101 PMC2860955

[b32] Gonzalez A. Rodriguez-Boulan E. (2009). Clathrin and AP1B: key roles in basolateral trafficking through trans-endosomal routes. FEBS Lett. 583, 3784–3795. 10.1016/j.febslet.2009.10.050 19854182 PMC4286365

[b33] Gravotta D. Deora A. Perret E. Oyanadel C. Soza A. Schreiner R. Gonzalez A. Rodriguez-Boulan E. (2007). AP1B sorts basolateral proteins in recycling and biosynthetic routes of MDCK cells. Proc. Natl. Acad. Sci. USA 104, 1564–1569. 10.1073/pnas.0610700104 17244703 PMC1785260

[b34] Hallworth R. Evans B. N. Dallos P. (1993). The location and mechanism of electromotility in guinea pig outer hair cells. J. Neurophysiol. 70, 549–558. 8410156 10.1152/jn.1993.70.2.549

[b35] Housley G. D. Marcotti W. Navaratnam D. Yamoah E. N. (2006). Hair cells – beyond the transducer. J. Membr. Biol. 209, 89–118. 10.1007/s00232-005-0835-7 16773496

[b36] Huang G. Santos-Sacchi J. (1993). Mapping the distribution of the outer hair cell motility voltage sensor by electrical amputation. Biophys. J. 65, 2228–2236. 10.1016/S0006-3495(93)81248-7 8298046 PMC1225954

[b37] Hunziker W. Harter C. Matter K. Mellman I. (1991). Basolateral sorting in MDCK cells requires a distinct cytoplasmic domain determinant. Cell 66, 907–920. 10.1016/0092-8674(91)90437-4 1909606

[b38] Kalinec F. Holley M. C. Iwasa K. H. Lim D. J. Kachar B. (1992). A membrane-based force generation mechanism in auditory sensory cells. Proc. Natl. Acad. Sci. USA 89, 8671–8675. 10.1073/pnas.89.18.8671 1528879 PMC49982

[b39] Leonova E. V. Raphael Y. (1997). Organization of cell junctions and cytoskeleton in the reticular lamina in normal and ototoxically damaged organ of Corti. Hear. Res. 113, 14–28. 10.1016/S0378-5955(97)00130-5 9387983

[b40] Liberman M. C. Gao J. He D. Z. Wu X. Jia S. Zuo J. (2002). Prestin is required for electromotility of the outer hair cell and for the cochlear amplifier. Nature 419, 300–304. 10.1038/nature01059 12239568

[b41] Lin S. Naim H. Y. Roth M. G. (1997). Tyrosine-dependent basolateral sorting signals are distinct from tyrosine-dependent internalization signals. J. Biol. Chem. 272, 26300–26305. 10.1074/jbc.272.42.26300 9334200

[b42] Mahendrasingam S. Katori Y. Furness D. N. Hackney C. M. (1997). Ultrastructural localization of cadherin in the adult guinea-pig organ of Corti. Hear. Res. 111, 85–92. 10.1016/S0378-5955(97)00091-9 9307314

[b43] Matter K. Hunziker W. Mellman I. (1992). Basolateral sorting of LDL receptor in MDCK cells: the cytoplasmic domain contains two tyrosine-dependent targeting determinants. Cell 71, 741–753. 10.1016/0092-8674(92)90551-M 1423629

[b44] Mellado Lagarde M. M. Drexl M. Lukashkina V. A. Lukashkin A. N. Russell I. J. (2008). Outer hair cell somatic, not hair bundle, motility is the basis of the cochlear amplifier. Nat. Neurosci. 11, 746–748. 10.1038/nn.2129 18516034

[b45] Ohno H. Tomemori T. Nakatsu F. Okazaki Y. Aguilar R. C. Foelsch H. Mellman I. Saito T. Shirasawa T. Bonifacino J. S. (1999). Mu1B, a novel adaptor medium chain expressed in polarized epithelial cells. FEBS Lett. 449, 215–220. 10.1016/S0014-5793(99)00432-9 10338135

[b46] Pietrini G. Suh Y. J. Edelmann L. Rudnick G. Caplan M. J. (1994). The axonal gamma-aminobutyric acid transporter GAT-1 is sorted to the apical membranes of polarized epithelial cells. J. Biol. Chem. 269, 4668–4674. 8308038

[b47] Rodriguez-Boulan E. Gonzalez A. (1999). Glycans in post-Golgi apical targeting: sorting signals or structural props? Trends Cell Biol. 9, 291–294. 10.1016/S0962-8924(99)01595-0 10407407

[b48] Rodriguez-Boulan E. Kreitzer G. Müsch A. (2005). Organization of vesicular trafficking in epithelia. Nat. Rev. Mol. Cell Biol. 6, 233–247. 10.1038/nrm1593 15738988

[b49] Russell I. J. Nilsen K. E. (1997). The location of the cochlear amplifier: spatial representation of a single tone on the guinea pig basilar membrane. Proc. Natl. Acad. Sci. USA 94, 2660–2664. 10.1073/pnas.94.6.2660 9122252 PMC20145

[b50] Santos-Sacchi J. (2003). New tunes from Corti's organ: the outer hair cell boogie rules. Curr. Opin. Neurobiol. 13, 459–468. 10.1016/S0959-4388(03)00100-4 12965294

[b51] Sugimoto H. Sugahara M. Fölsch H. Koide Y. Nakatsu F. Tanaka N. Nishimura T. Furukawa M. Mullins C. Nakamura N. (2002). Differential recognition of tyrosine-based basolateral signals by AP-1B subunit mu1B in polarized epithelial cells. Mol. Biol. Cell 13, 2374–2382. 10.1091/mbc.E01-10-0096 12134076 PMC117320

[b52] Vicidomini G. Moneron G. Han K. Y. Westphal V. Ta H. Reuss M. Engelhardt J. Eggeling C. Hell S. W. (2011). Sharper low-power STED nanoscopy by time gating. Nat. Methods 8, 571–573. 10.1038/nmeth.1624 21642963

[b53] Weisz O. A. Rodriguez-Boulan E. (2009). Apical trafficking in epithelial cells: signals, clusters and motors. J. Cell Sci. 122, 4253–4266. 10.1242/jcs.032615 19923269 PMC2779128

[b54] Yu N. Zhu M. L. Zhao H. B. (2006). Prestin is expressed on the whole outer hair cell basolateral surface. Brain Res. 1095, 51–58. 10.1016/j.brainres.2006.04.017 16709400 PMC2548272

[b55] Zheng J. Shen W. He D. Z. Long K. B. Madison L. D. Dallos P. (2000). Prestin is the motor protein of cochlear outer hair cells. Nature 405, 149–155. 10.1038/35012009 10821263

[b56] Zheng J. Du G. G. Matsuda K. Orem A. Aguiñaga S. Deák L. Navarrete E. Madison L. D. Dallos P. (2005). The C-terminus of prestin influences nonlinear capacitance and plasma membrane targeting. J. Cell Sci. 118, 2987–2996. 10.1242/jcs.02431 15976456

[b57] Zheng L. Zheng J. Whitlon D. S. García-Añoveros J. Bartles J. R. (2010). Targeting of the hair cell proteins cadherin 23, harmonin, myosin XVa, espin, and prestin in an epithelial cell model. J. Neurosci. 30, 7187–7201. 10.1523/JNEUROSCI.0852-10.2010 20505086 PMC2989820

