# Supplementary material for: Tyrosine motifs are required for prestin basolateral membrane targeting
Source: Biol Open. 2015 Jan 14;4(2):197–205. doi: 10.1242/bio.201410629 (PMC4365488; doi:10.1242/bio.201410629)
Supplement: Supplementary Material [file supp_4_2_197__index.html]

Tyrosine motifs are required for prestin basolateral membrane targeting — Supplementary Material 

# Tyrosine motifs are required for prestin basolateral membrane targeting

## bio.201410629 Supplementary Material

**Files in this Data Supplement:**

- Supplementary Material - Yifan Zhang et al. doi: 10.1242/bio.201410629
