# Supplementary material for: Tyrosine motifs are required for prestin basolateral membrane targeting
Source: Biol Open. 2015 Jan 14;4(2):197–205. doi: 10.1242/bio.201410629 (PMC4365488; doi:10.1242/bio.201410629)
Supplement: Supplementary Material [file supp_bio.201410629_bio.201410629-s1.pdf]

Biology Open (2015) 000, 1–9 doi:10.1242/bio.201410629

Supplementary Material
Yifan Zhang et al. doi: 10.1242/bio.201410629

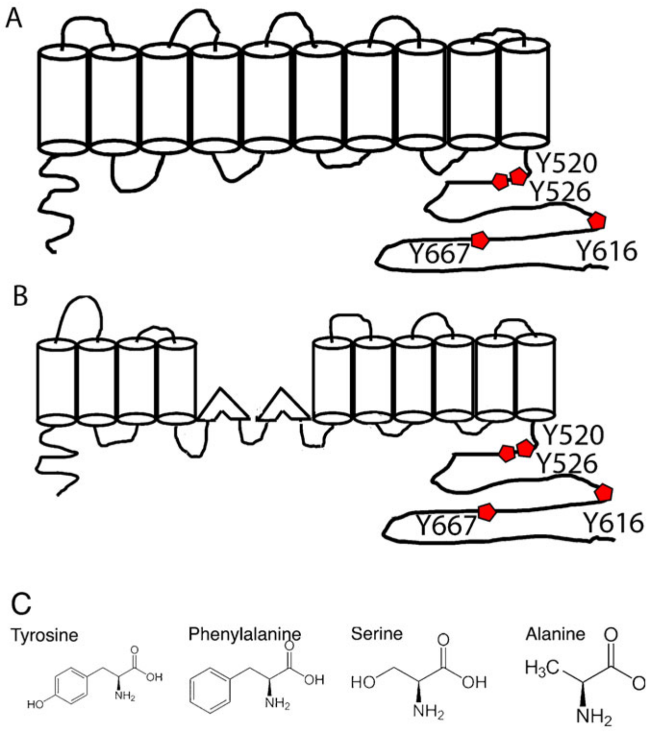

Fig. S1. A cartoon of the localization of individual C-terminal tyrosine residues within the 10 (A) and 12 (B) transmembrane models of prestin are shown. (C) The structure of individual residues including phenylalanine, serine and alanine used to substitute for individual tyrosine residues are compared.

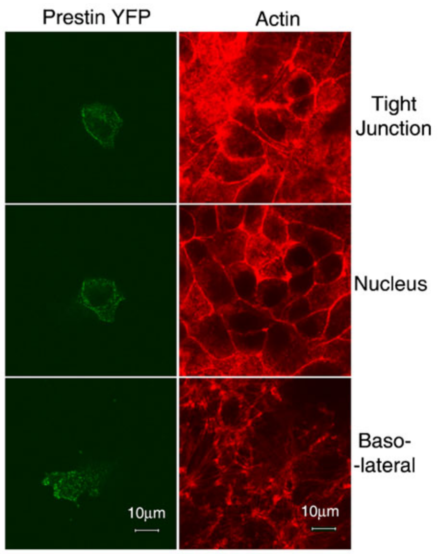

Fig. S2. LLC-PK cells show deficient basolateral targeting of prestin YFP. Confluent polarized LLC-PK1 cells were transfected with prestin YFP (green) and imaged by confocal imaging after fixation 72 hours later. Shown are serial X-Y sections along the z axis of a transfected cell (at the level of the tight junction, nucleus and basolateral surface). The actin cytoskeleton was stained with Phalloidin Alexa 647 (red). There is reduced targeting of prestin at the basolateral surface of the cell.

Table S1. Parameters of non linear capacitance in prestin and specific tyrosine mutants

	Q_{sp} (fC/pF)	V_h (mV)	z	n
Prestin	10.73+/−1.02	−115.44+/−4.32	0.78+/−0.02	7
Y667F	17.57+/−3.56	−149.18+/−8.34	0.82+/−0.02	5
Y667Q	0.92+/−0.24	−103.39+/−8.73	0.81+/−0.05	5
Y520F	2.75+/−0.75	−97.62+/−6.03	0.81+/−0.03	6

Measures of NLC in different mutants of prestin at Y667 and Y520 are compared. Other mutations at these sites did not show NLC. Note that substitution of Y520 and Y667 with a phenyl alanine residue resulted in divergent effects on Vh with the former causing a mild depolarization shift while the latter caused a marked hyperpolarization shift. The latter was also associated with an increase in Qsp.
